# Infant Sleeping Environment in South-Eastern Nigeria (Sleeping Place and Sleeping Position): A Preliminary Survey

**DOI:** 10.1155/2009/283046

**Published:** 2009-09-13

**Authors:** Ngozi S. Ibeziako, Roland Chidi Ibekwe, Bede C. Ibe

**Affiliations:** ^1^Department of Paediatrics, College of Medicine, University of Nigeria, Enugu Campus, Nigeria; ^2^Department of Paediatrics, College of Health Sciences, Ebonyi State University, Abakaliki, Ebonyi State, Nigeria

## Abstract

*Objective*. To determine infant sleeping position/place and the factors associated with them in South-eastern Nigeria. *Methods*. this is a cross-sectional study on infant sleeping environment. Subjects were the mother/ infant pairs that attended the well baby clinics at the Institute of Child Health of the University of Nigeria Teaching Hospital, Enugu (ICH-UNTH), Mother of Christ Specialist Hospital (MCSH), Enugu and the Ebonyi State University Teaching Hospital (EBSUTH), Abakaliki. *Results*. Lying on the side was the most common (51.1%) and the least stable sleeping position. Only 36.6% of infants who slept in that position were likely to be found in the same position the following morning; lying supine was the most stable (74.1%). The difference in stability of sleeping positions was statistically significant (*P* < .01). Twenty six point seven percent of the mothers routinely lay their infants in prone position. On logistic regression, maternal parity was the only factor that was predictive of nonprone sleeping position (*P* = .01). Bed sharing, though common (66.9%), was more among the experienced (*P* = .03) and less educated mothers (*P* < .01). *Conclusion*. There is a high level of prone sleeping position and bed sharing among infants in this study site. The potential consequences of these are unclear. There is therefore a need to conduct local studies to clarify its implication.

## 1. Introduction

The implications and impact of child sleeping place and position have generated interest in developed countries in the last decade because merging evidence seems to suggest a causal relationship with Sudden Infant Death Syndrome (SIDS), a leading cause of postneonatal infant mortality [1, 2]. Factors commonly linked with the increased risk of SIDS include black race, low birth weight, young maternal age, low maternal education, high parity, and late or no antenatal care [2]. This is in contrast to the situation in developing countries where the above risk factors are “native” due to socioeconomic demographics and are usually associated with the leading causes of infant morbidity and mortality, which are mainly infections and infestations [3, 4]. 

Recent epidemiological studies in New Zealand, United Kingdom, Australia, and USA that focused on the infant sleeping environment which are “modifiable” found a strong association between prone sleeping position and SIDS [1, 5, 6]. In 1992, the American Academy of Pediatrics (AAP) Task force on infants' sleeping position and SIDS recommended that all healthy full-term infants should be placed either lateral (on the side) or supine (on the back) to sleep [7]. This was followed by a public education campaign on “Back to Sleep,” an intervention that resulted in 66% reduction in prone sleeping and 33% decline in SIDS rate [7].

The infant sleeping place has also elicited interest since a case control study in England found that bed sharing (bedding-in with mother or other adults) was more common in cases of SIDS than in controls [8]. Similarly, a nationwide study in New Zealand found a twofold increase in the risk of SIDS associated with bed sharing [9]. However, Klonoff-Cohen and Edelstein [10] in another case control study in California, USA, did not find any significant relationship between routine bed sharing and SIDS.

Although SIDS is not a leading cause of postneonatal mortality, there has been little interest on this topic by researchers in Africa. In view of the high infant mortality rate of 112/1000 livebirth in this country [4], any scientifically proved intervention to reduce it needs to be validated. There is, to our knowledge, no information on the infant sleeping environment in Nigeria. This study is therefore undertaken as a preliminary survey to determine the choice of infants' sleeping places and positions in this country. It is hoped that the finding from this study would contribute in forming the basis for health education of mothers on safer infant sleeping environment.

## 2. Subjects and Methods

### 2.1. Study Design

This cross-sectional study was carried out between March and May 2006 among mother-infant pairs attending the well baby clinics at the Institute of Child Health of University of Nigeria Teaching Hospital, Mother of Christ Specialist Hospital, both in Enugu, and the Ebonyi State University Teaching Hospital, Abakaliki. These centres were chosen because they enjoy large patronage, and the authors have working interest in them. Enugu is the capital city of Enugu State while Abakaliki is the capital of Ebonyi State, both in South-East of Nigeria. The populations of Enugu and Abakaliki are 308 200 and 235 000, respectively. The inhabitants are mainly Ibos.

### 2.2. Sample Size and Sampling Technique

Literature search showed no local prevalence rates of either sleeping position or sleeping place practices. We therefore assumed a population prevalence rate of bed sharing to be 50% to allow for the widest variance possible. Using the formula for sample size determination (*N* = *Z*_2_ *P* *Q*/*d*_2_), a sample size of 382 was obtained. This was increased to 500 to include attrition rate of 30%.

### 2.3. Subjects

These were mother-infant pairs with children aged 1–52 weeks and who were brought by their mothers for immunization and growth monitoring at the clinics. Only mothers who gave informed consent and had singletons were interviewed. Excluded from the study were those who were delivered pre term, or those whose babies were admitted into the newborn special care unit for any reason. Children with identifiable chronic conditions like congenital heart disease, cerebral palsy, or orofacial anomaly were also excluded.

### 2.4. Study Tool

The study tool was a structured pretested interviewer administered questionnaire on routine sleeping positions, classified as “on the stomach with face down,” “on the stomach with face to the side” (these two sleeping position were considered as prone positions), “on the back (supine) or on the side (lateral).” To determine the stability of the different sleeping positions, the position the child was laid to sleep the previous night, classified as any of the above position, and the position the child was found on awakening the following morning was also sought. Information on the routine sleeping place for the infant was noted, that is, a crib, pram, cradle, parents' or other persons' bed, couch, floor, or any other place. Bed sharing was defined as routinely sharing a bed with a specified person, including the mother, father, other relatives (sibling, aunt, grand parent, etc.) or baby sitter. Demographic information on maternal age, highest educational attainment, occupation, parity (those with 2 or more children were classified as “experienced” while those with only one were classified as “inexperienced”), and child's sex and age was also obtained.

### 2.5. Ethical Approval

The Ethical Committees of the University of Nigeria Teaching Hospital Enugu, the Ebonyi State University Teaching Hospital Abakaliki, and the authorities of the Mother of Christ Specialist Hospital Enugu approved the study.

### 2.6. Statistical Analysis

Analysis was done using SPSS statistical package version 11.0 using tables and percentages. Means and standard deviations were determined as appropriate, and differences in proportions were tested for statistical significance using the chi-square test. Weighted Kappa was used to compare the concordance between the infants' sleeping and waking positions, and logistic regression was used to determine factors that were predictive of prone sleeping position. Significance level was set at *P* < .05, with 95% confidence level.

## 3. Results

### 3.1. Demographic Characteristics

The mother/infant pairs were recruited consecutively as they attended clinics at the 3 sites until the sample size was obtained. The 3 sites contributed 215, 171, and 114 from ICH-UNTH, MCSH, and EBSUTH, respectively. Four hundred and eighty mother/infant pairs were analyzed. Twenty were not included in the analysis due to incomplete data. Table 1 shows the demographic characteristics of the mothers and infants that participated in this study. Three hundred and twenty eight mothers were aged between 21 and 30 years while the age of 115 mothers was between 31 and 40; the two age categories constituted 92.7% of the mothers. Three hundred and ninety five (83.2%) mothers had at least secondary education. Petty trading and full-time homemaking were the two most common occupations among the mothers. Two hundred and ninety three mothers (61.3%) reported their parity. The mean number of deliveries per mother was 3.29 ± 1.61. Of these, 26 (8.9%) were primipara; others had two or more infants. Thus 267 (91.1%) of the mothers were experienced. 

There were 246 males (51.3%) and 233 females (48.6%), M : F = 1 : 1.06. The mean age of the infants was 14.29 weeks ± 12.82, and 313 (65.2%) of them were aged 1–12 weeks.

### 3.2. Infants' Sleeping Characteristics

#### 3.2.1. Sleeping Place

Three hundred and twenty one (66.9%) infants routinely slept with their parents or siblings in the same bed. Table 2 shows the association between bed sharing and various demographic variables of mothers (caregivers) and infants. Bed sharing was more common among experienced mothers (*P* = .03) and less educated mothers (*P* < .01). Older infants were also more likely to share bed (*P* = .03). Maternal age and baby's sex were not significantly associated with baby's sleeping place.

#### 3.2.2. Sleeping Position

The most common sleeping position adopted for the babies was lying on the side. Out of the 452 sleeping positions recorded, 234 (51.8%) routinely lie their infants on their side, 121 (26.7%) laid prone, and 97 (21.5%) usually lie supine.

Table 3 shows that lying by the side was the least stable position as only 33.6% of children that were laid by the side the previous night were found in that position the following morning. Majority (57.96%) woke up in supine position. The most stable sleeping position was the supine position, as 61.3% of the infants that were laid in this position at night were found in the same position the following morning. This relationship is statistically significant (Kappa = 0.223, *P* < .01).

Maternal experience (*P* < .01) was the only factor that was predictive of nonprone sleeping position on logistic regression analysis as shown in Table 4. Maternal age and education, and infants' sex and sleeping place had no significant predictive influence on infants' sleeping position.

## 4. Discussion

A quarter of the mothers laid their infants prone in this study. This is lower than the prelevel “back to sleep” in the USA [7] and Netherlands [1], and still falls short of the post target “back to sleep” of less than 10% [7]. The instability associated with sleeping on the side was also observed in this study as majority of children who were put to bed on the side either woke up supine or prone [1, 5–7]. Mothers are said to lay their infants by the side for fear of aspiration and choking in their vomit. Though this has not been confirmed by research evidence, studies have shown that health education can modify mothers' attitude regarding infants' sleeping position [7, 11]. Mothers are also said to lie their infants by the side because it facilitates breast feeding at night. The proportion of mothers that breast feed and its relationship with infant sleeping position was not explored in this study.

Bed sharing is common among the respondents in this study. The rate of 67% is higher than the rate of 12.8%–48% reported in the USA [12, 13]. This difference could be due to cultural practices in Nigeria and the Baby Friendly Hospital Initiative (BFHI) which promotes exclusive breast feeding, rooming-in, and bedding-in [14]. In the USA, bed sharing was found to be independently associated with Black race and breastfeeding [12, 13]. However, black women in the USA were found to have very low breastfeeding rates [15]. There has been recent controversy about the merits and demerits of bed sharing. While advocates postulate that it promotes parent-infant bonding, facilitates breast feeding, and by promoting maternal vigilance also reduces the risk of SIDS [16], critics discourage the practice because it was found to increase the risk of SIDS especially among a subset of mothers who smoke [6–8]. The role of maternal smoking was not explored in this study. However, in developing countries like Nigeria where the prevalence of malnutrition, diarrhea disease, and poverty remains high, activities that promote breast feeding should be encouraged [13]. 

We observed that “experienced” (multiparous) mothers tend to cosleep with their infants. Similar findings have been reported in America and New Zealand where it was noted that mothers with more children were also likely to be less educated, younger, and poorer and lived in less spacious accommodation [9, 10]. The reason for this finding was considered mainly economic. It is to be noted that though the experienced mothers in this study were not younger or less educated than the inexperienced ones, it is likely that they were more confident and felt they are unlikely to compromise their infants if they sleep with them. The adequacy of the living space was not explored in this study. Unlike in New Zealand [9] where bed sharing wanes as the infants grow older, we found that older infants were more likely to share beds with their parents. The reason for this is not clear.

The more educated mothers in this study were less likely to share bed with their infants. This is similar to the findings in developed countries [9, 10]. The reason for this is most likely economic since they are more likely to afford bigger houses with space for the infants. The study population appears to consist of relatively well-educated mothers. This is rather surprising, since the study sites are Government and mission health facilities whose services are cheap and are supposed to be patronized by the poor. The reason may be because the infant welfare clinics offer preventive and health promotion services which educated mothers are more likely to appreciate. 

Earlier reports seem to suggest that bed sharing promotes nonprone sleeping [15] but we found no association between sleeping position and bed sharing. While prone sleeping was significantly associated with multiparity in the New Zealand [9] and USA [10] studies, the reverse was documented in our study. This difference could be cultural because women that are likely to have more children in developed countries were likely to be relatively poorly educated, single mothers with poor access to health facilities. In Nigeria, multiparity is not necessarily associated with these factors. 

The high non-response rate of 39.0% on “parity” can be explained on the cultural perception among the Ibos that children should not be counted. In the Ibo culture, there is a belief that once a child is counted, then that child is unlikely to live. This perception was supported by the high infant and under-five mortality among the Ibos. Due to this belief, children were only counted when they survive through their fifth birthday.

We conclude that there is a high level of prone sleeping position and bed sharing among mother-infant pairs in this study site. Though the potential consequences of these are unclear, the known adverse consequences of prone sleeping positions should be emphasized to mothers while further local studies to clarify its' implication is recommended.

## Figures and Tables

**Table 1:** Distribution of maternal and infant demographics.

Maternal Age	Frequency	Percentage
16–20	21	4.4
20–30	328	68.3
31–40	115	24.0
>40	4	0.8
No response	12	2.5

Total	480	100.0

Maternal Educational Attainment		
University/polytechnic	215	44.8
Secondary Education	180	37.5
Primary Education	73	15.2
No Formal Education	7	1.5
No Response	5	1.0

Total	480	100

Maternal Occupations		
Senior Civil Servant	84	17.5
Junior Civil Servant	53	11.04
Petty Trader	103	21.5
House Wife	120	25.0
Student	20	4.16
Self employed	78	16.3
Corporate office	5	10
No response	17	3.5

Total	480	100

Number of Delivery by Mothers		
1.	26	5.4
1–4	194	40.4
>5	73	15.2
No response	187	39.0

Total	480	100

Age of Infant (Wks)		
1–12	314	65.4
13 –24	70	14.6
25 –36	66	13.8
37–52	29	6.0
No response	1	0.2

Total	480	100.0

Sex of Infants		
Male	246	51.3
Female	233	48.5
No response	1	0.2

Total	480	100.0

**Table 2:** Association between infant sleeping place and some maternal and infant variables.

Variables	Infant Sleeping Place		Total	*P*
	Bed sharing	No Bed sharing		
Mothers Age.(Yrs)				
16–20	12	8	20	0.69
21–30	228	91	319	
31–40	73	37	110	
>40	2	2	4	

Maternal Experience				
Inexperience	13	12	25	0.03
Experienced	186	70	256	

Maternal Education				
University/Polytechnic	121	89	210	<0.01
Secondary/Vocational	134	37	171	
Primary	62	10	72	
No Formal Education	3	3	6	

Infant Age. (Wks)				
1–12	197	103	300	0.03
13–24	48	20	68	
25–36	53	10	63	
37–52	23	5	28	

Infant Sex				
Male	166	72	238	0.93
Female	155	66	221	

**Table 3:** Infants' sleeping position at night and position found on waking in the morning. Kappa = 0.22, *P* < .01.

Infants' sleeping position	Infants' waking position			Total
	Prone *N* (%)	Supine *N* (%)	Side *N* (%)	*N* (%)
Prone	71 (59.2)	28 (23.3)	21 (17.5)	120 (100)
Supine	9 (9.7)	57 (61.3)	27 (29.0)	93 (100)
Side	19 (8.4)	131 (58.0)	76 (33.6)	226 (100)

Total	99 (22.6)	216 (49.2)	124 (28.2)	439 (100)

**Table 4:** Logistic regression analysis of variables with predictive influence on prone sleeping position.

Variables	*B*	S. E.	Wald	*R*	Exp (*B*)	*P*
Mothers Age	0.22	0.16	1.99	0.00	1.25	0.16
Maternal Experience	−1.16	0.45	6.53	−0.12	0.31	0.01
Mothers Educational Status	−0.32	0.21	2.46	−0.04	0.72	0.12
Age Categories (Weeks)	−0.30	0.17	3.35	−0.07	0.74	0.07
Sex	−0.51	0.30	2.94	−0.06	0.60	0.09
Babies Sleeping Place	−0.19	0.33	0.31	0.00	0.83	0.58
Constant	2.39	1.27	3.54			0.06
